# Immune Effect of Active Components of Traditional Chinese Medicine on Triple-Negative Breast Cancer

**DOI:** 10.3389/fphar.2021.731741

**Published:** 2021-12-03

**Authors:** Wenjie Zhao, Jinhua Liu, Yaqun Li, Zichao Chen, Dongmei Qi, Zhen Zhang

**Affiliations:** ^1^ Innovation Research Institute of Traditional Chinese Medicine, Shandong University of Traditional Chinese Medicine, Jinan, China; ^2^ College of Chinese Medicine, Shandong University of Traditional Chinese Medicine, Jinan, China; ^3^ Experimental Center, Shandong University of Traditional Chinese Medicine, Jinan, China

**Keywords:** triple-negative breast cancer, traditional Chinese medicine, herbal remedies active ingredients, tumor immunity, anti-tumor

## Abstract

Triple-negative breast cancers are heterogeneous, poorly prognostic, and metastatic malignancies that result in a high risk of death for patients. Targeted therapy for triple-negative breast cancer has been extremely challenging due to the lack of expression of estrogen receptor, progesterone receptor, and human epidermal growth factor receptor 2. Clinical treatment regimens for triple-negative breast cancer are often based on paclitaxel and platinum drugs, but drug resistance and side effects from the drugs frequently lead to treatment failure, thus requiring the development of new therapeutic platforms. In recent years, research on traditional Chinese medicine in modulating the immune function of the body has shown that it has the potential to be an effective treatment option against triple-negative breast cancer. Active components of herbal medicines such as alkaloids, flavonoids, polyphenols, saponins, and polysaccharides have been shown to inhibit cancer cell proliferation and metastasis by activating inflammatory immune responses and can modulate tumor-related signaling pathways to further inhibit the invasion of triple-negative breast cancer. This paper reviews the immunomodulatory mechanisms of different herbal active ingredients against triple-negative breast cancer and provides an outlook on the challenges and directions of development for the treatment of triple-negative breast cancer with herbal active ingredients.

## Introduction

Breast cancer is a serious threat to women’s life and health safety, especially triple-negative breast cancer (TNBC). The main feature of the disease is the lack of expression of estrogen receptor, progesterone receptor, and human epidermal growth factor receptor 2 (HER2) (Pareja and Reis-Filho, 2018). Neither endocrine therapy nor conventional targeted therapy is the best treatment (Goto et al., 2018). Chemotherapy is still the mainstay of advanced TNBC with paclitaxel/anthracycline-containing agents as the chemotherapy of choice, and platinum-based agents in combination with paclitaxel as an effective alternative adjuvant chemotherapy option for patients with operable TNBC (Coates et al., 2015; Yu et al., 2020). But these drugs were not developed as precise treatments based on the genetic and hereditary characteristics of TNBC itself, although the above regimens have resulted in prolonged survival cycles, the toxic effects are heavy and still not tolerated by some patients (Goto et al., 2018). Without other effective approaches, it may mean a poor prognosis. There is an urgent need to find a new effective anti-TNBC drug with few side effects (Chu et al., 2003).

**FIGURE 1:**
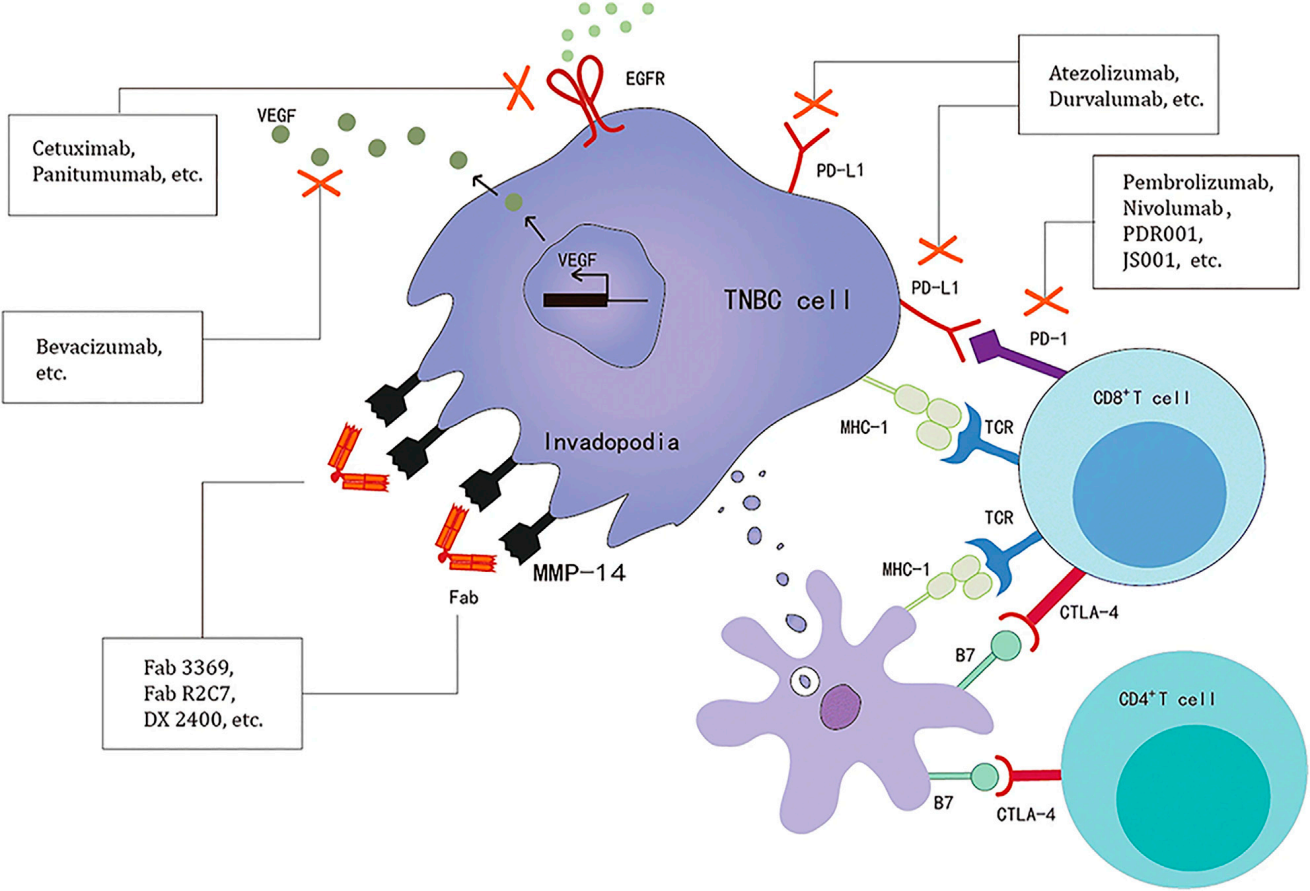
TNBC-related immune drug targets (Li et al., 2018).

TNBC is a kind of highly malignant cancer. It’s evolution and drug resistance remain the greatest challenges to disease treatment. Since targeted therapies are less effective against TNBC, immunotherapy may benefit patients. Immune-related drug targets are currently used in the clinical treatment of TNBC patients (Figure 1) (Li et al., 2018a). Paul Ehrlich, who first proposed the doctrine of immune surveillance in 1909, suggested that abnormal immune function might contribute to the development of tumors (Ehrlich., 1909). The first immune detection site to be used in medical practice was cytotoxic T lymphocyte-associated antigen-4 (CTLA-4), discovered in 1987 (Brunet et al., 1987). James Allison’s group first demonstrated in experiments in mice that the use of CTLA-4 antibodies could enhance immunity and suppress tumor development in 1996 (Leach et al., 1996). Programmed cell death-1 (PD-1) was discovered in 1992, and studies targeting the immune checkpoints CTLA-4, PD-1, and programmed cell death-Ligand 1 (PD-L1) have led to breakthroughs in a variety of cancer types. PD-1 inhibitors have brought the treatment of malignant tumors into a new era of immunotherapy (Ishida et al., 1992; Zhang et al., 2021). PD-1, in combination with PD-L1, can transmit inhibitory signals and help tumors undergo immune escape. Approximately 20% of TNBC express PD-L1 (Mittendorf et al., 2014), targeting PD-L1 therapy in TNBC patients with positive PD-L1 expression was found to prolong the survival of such patients (Romero, 2019).

The database for Traditional Chinese Medicine on Immuno-Oncology contains 400 tumor immune targets from different literature and the corresponding 126,972 ligand molecules, demonstrating the active role of traditional Chinese medicine (TCM) in immune function (Liu et al., 2020). TCM works by modulating immune cells in the body, such as T lymphocytes, bursa dependent lymphocytes, natural killer cells, and macrophages cells, which can exert anti-tumor immune effects (Wang et al., 2020). There are many studies on the combined application of TCM with targeted drugs or immunotherapy, with the main purpose of improving efficacy, reversing drug resistance, reducing adverse effects, and prolonging the survival of patients (Chu et al., 2003; Liu et al., 2019a). Therefore, this paper focuses on the summary of different active ingredients of TCM in immunotherapy for TNBC.

### Immune Effect of TCM Containing Alkaloids on TNBC

Plant alkaloids play an anti-cancer effect in cancer treatment. By regulating immune function, inhibiting angiogenesis, and inducing cell apoptosis (Efferth and Oesch, 2021). Tetrandrine has a wide range of antitumor effects and can inhibit MDA-MB-231 cell proliferation and induce cellular autophagy by inhibiting phosphatidylinositol-3-kinase (P13K), protein kinase B (AKT), mammalian target of rapamycin (mTOR) pathway (Liu et al., 2016; Guo and Pei, 2019). Reactive oxygen species (ROS) are a class of molecules produced during oxidative stress in the body, which are closely related to tumor immune tolerance and play a key role in immune monitoring (Bailly, 2020; Hu et al., 2021). ROS-triggered small ubiquitin-like modifier (SUMO)-specific protease 3 accumulation is involved in Treg cell-mediated immunosuppression (Yu et al., 2018). In addition to ROS, activation of the Ras, mitogen-activated protein kinase (MAPK) pathway may promote tumor immune evasion in TNBC (Loi et al., 2019). Sinomenine induced apoptosis in MDA-MB-231 cells by upregulating the MAPK pathway and by increasing intracellular ROS production. It also decreased the expression of IL-8, chemokine receptor 2(CXCR2) in MDA-MB-231 cells. Inhibited the activation of nuclear factor kappa-B (NF-κB) and sonic hedgehog signaling pathways in TNBC lung metastasis. (Li et al., 2014; Song et al., 2018; Zhang et al., 2019).

Berberine increased the release of lactate dehydrogenase in MDA-MB-231 cells in a dose-dependent manner, which led to a significant decrease in the secretion of IL-1α, IL-1β, IL-6, and TNF-α at the same time. Synergistic treatment with berberine and lipopolysaccharide significantly reduced the expression of TNF-α and IL- 6 (Zhao et al., 2017; Kim et al., 2018; Lin et al., 2019; Yao et al., 2019; Yao et al., 2019; Zhao and Zhang, 2020). High mobility group box-1(HMGB1) protein is an endogenous member of the proteome called danger-associated molecular pattern that promotes proliferation and survival of a variety of immunosuppressive cells. Synergistic effects of theophylline and berberine induce cell cycle arrest and poly ADP-ribose polymerase (PARP), HMGB1 protein and b-cell lymphoma 2 family-mediated apoptosis in MDA-MB-231 cells (Wild et al., 2012; Hashemi-Niasari et al., 2018).

Cisplatin resistance is associated with breast cancer susceptibility genes. The PARP inhibitor olaparib induces CD8^+^ T cell activation *in vivo* (Pantelidou et al., 2019; Pettitt et al., 2020). Triptolide downregulates PARP1 levels and inhibits BT549 as well as MDA- MB-231 cell growth, mainly by interfering with X-ray repair cross-complement 1, PAPR1-mediated base excision repair, thereby sensitizing TNBC to cisplatin (Zhang et al., 2019). Both Celastrol and Triptolide inhibit mammosphere formation in MDA-MB-231, BT20 progenitor cells. It inhibited the expression of stem cell marker proteins doublecortin-like kinase 1, aldehyde dehydrogenase, and CD133 (Prominin-1) (Ramamoorthy et al., 2021).

In addition to the above TCM containing alkaloids, such as aconitine, betaine, ephedrine, and evodiamine, which also have antitumor effects (Ma and Wink, 2008; Du et al., 2013; Hyuga et al., 2013; Tan et al., 2020; Zhang et al., 2021), but the immune mechanism of action on TNBC still deserves further investigation.

### Immune Effect of TCM Containing Phenolic Compound on TNBC

During tumor progression, tumor cells continuously interact with the microenvironment and mediate immune tolerance. Polyphenols, the active ingredients of TCM, can inhabit the tumor microenvironment (Hui and Chen, 2015; Bian et al., 2020). The ability of breast cancer stem cells to self-renew is closely related to disease progression, and high expression of stem cell estrogen receptor alpha-36 (ER-α36) (Deng et al., 2014). Epigallocatechin-3-gallate is a type of catechin extracted from green tea, effectively inhibited the growth of tumor stem, progenitor cells in MDA-MB-231 and MDA-MB-436 cells and reduced the expression of ER-α36 in these cells (Pan et al., 2016). F-Box and WD repeat domain containing 7 (FBXW7) can inhibit tumor development by acting on the tumor microenvironment (Yumimoto et al., 2015). Honokiol downregulates miR-188-5p *via* FBXW7, c-Myc signaling, enhances the sensitivity of human breast cancer to doxorubicin. Honokiol exerts anti-proliferative activity during cell cycle arrest in G0/G1 phase and inhibits the proliferation of MDA-MB-231 cells by suppressing the c-Src, epidermal growth factor receptor (EGFR)-mediated signaling pathway. However, due to the limited oral bioavailability of honokiol, the use of vitamin E polyethylene glycol succinate in nanocapillary formulations enhances the anti-cancer effect of *in situ* TNBC (Park et al., 2009; Godugu et al., 2017; Yi et al., 2021). The NF-κB pathway, a key regulator of the immune response, is frequently dysregulated in cancer (Tegowski and Baldwin, 2018). Magnolol significantly inhibited the activity of highly invasive MDA-MB-231 cells and down-regulated the expression of matrix metallopeptidase 9 (MMP-9), as well as the transcriptional activity of NF-κB and the deoxyribonucleic acid (DNA) binding of NF-κB to the MMP-9 promoter (Liu et al., 2013).

The flavonoids found in TCM have a variety of biological properties, including anti-inflammatory and anti-angiogenic properties, play an immune role by influencing immune organs, cellular immunity, non-specific immunity, and immune-related signal transduction pathways. (Casagrande and Darbon, 2001; Bagli et al., 2004; Ullmannova and Popescu, 2007; Maleki et al., 2019). Puerarin inhibited the migration, invasion and adhesion of lipopolysaccharide-stimulated MDA-MB-231 cells by inhibiting the NF-κB pathway and extracellular regulated protein kinases (ERK) phosphorylation (Liu et al., 2017). However, due to poor water solubility and low bioavailability of puerarin, new puerarin nanoemulsions were developed to induce changes in the immune microenvironment of 4T1 cells, improving the therapeutic efficiency of α-PD-L1 in TNBC models and downregulating intra-tumor ROS (Xu et al., 2020). Licorice flavonoids promote ROS production, increase endogenous and exogenous apoptotic pathways, promote PARP-1 activation in MDA-MB-231 cells, and induce DNA damage. It regulates autophagy and blocks cell growth by inhibiting phosphorylation of AKT and MAPK signaling pathways, regulating the expression of E-cadherin and vimentin (Huang et al., 2019) (-)-Epigallocatechin 3-gallate is a natural polyphenol extracted from green tea, and a series of derivatives were synthesized to improve the pharmacological structure, and all of these compounds were found to exhibit moderate to high cytotoxicity against MDA-MB-231 cells (Crous-Masó et al., 2018).

TCM such as paeonol and curcumin also contain phenolic active ingredients and play an important role in the anti-tumor process (Cai et al., 2014; Hu et al., 2019), which still need to be further explored in the process of immunotherapy for TNBC.

### Immune Effect of Saponins on TNBC

Saponins have pharmacological activities such as antioxidant, anti-inflammatory, and antitumor. The inhibitory effect of ginseng on breast cancer cell growth is mainly through transcriptional upregulation of the cell cycle protein-dependent kinase inhibitors p21 and p53 (Block and Mead, 2003; AL Shabanah et al., 2016). The combination of ginsenoside panaxatriol with paclitaxel inhibited the activation of interleukin-1 receptor-associated kinase 1(IRAK1), NF-κB and ERK1/2, leading to the inhibition of inflammatory factors, and cancer stem cell-related genes expression was downregulated and also inhibited the invasive ability of MB231-PR (paclitaxel-resistant) cells, reducing stem cell properties and resensitizing TNBC paclitaxel-resistant cells to paclitaxel by inhibiting the IRAK1/NF-κB and ERK pathways (Wang et al., 2020). In contrast, ginsenoside Rg1 induces apoptosis through ROS production and inhibits the development of triple negative breast cancer cells (Chu et al., 2020).

Ginsenoside Rh2 induces apoptosis, reverses abnormal differentiation of tumor cells, and resists tumor metastasis, and can be used in combination with chemotherapeutic drugs to increase the effectiveness and reduce the toxicity. Biochanin A combined with ginsenoside Rh2 has a synergistic effect on the proliferation of MDA-MB-231 and MCF-7 cells (Ren et al., 2018). Ginsenoside Rg3 in combination with paclitaxel treatment inhibited NF-kB activation, decreased NF-kB p65 and B-cell lymphoma-2 (Bcl-2) protein expression, and increased Bcl2-associated X and Caspase-3 protein expression, thus promoting the toxicity of paclitaxel on MDA-MB-231, MDA-MB453, and BT-549 cells (Yuan et al., 2017). After analysis using The Cancer Genome Atlas database identified a role for C-X-C motif chemokine ligand 12 (CXCL12), C-X-C motif chemokine receptor 4 (CXCR4) signaling pathway in oncogenic CD8^+^ T cells from human breast cancer, CXCR4 inhibition could enhance the efficacy of immunotherapy in the treatment of metastatic breast cancer (Chen et al., 2019). At doses without significant cytotoxicity, ginsenoside Rg3 treatment resulted in weaker CXCR4 staining in MDA-MB-231 cells, reduced the number of cells migrating during CXCL12-induced chemotaxis, and significantly reduced the number of CD44 ^high^/CD24 ^low^ in MDA-MB-231 cells with therapeutic potential for targeting breast cancer stem cells, possibly through classical mitochondria-dependent caspase activation to induce apoptosis in MDA-MB-231 cells (Chen et al., 2011; Kim et al., 2013; Oh et al., 2019).

Murine double minute2 (MDM2) controls the stability of STAT5 in CD8^+^ T cells and targeting p53-MDM2 interactions is essential for effective antitumor immunity. Polyphyllin D can inhibit MDA-MB-231 cell proliferation by decreasing the expression levels of MDM2, murine double minute x, and mutant p53 and inducing cell cycle arrest by upregulating the expression of MDM2 downstream proteins p21 and p27 (Kong et al., 2016; Zhou et al., 2021). RELT-like protein 2 (Rell2) is a direct target of mircoRNA-18a, and polyphyllin VI increased Rell2 expression and impaired the viability of 4T1 and MDA-MB-231 cells (Wang et al., 2019). Timosaponin AIII, a steroidal saponin, triggers DNA damage in breast cancer, activates the ataxia telangiectasia mutated, cell cycle checkpoint kinase 2 and p38 MAPK pathways, which in turn induces G2/M phase block and MDA-MB-231 apoptosis, and downregulates cell division cycle B1, cell division cycle 2, and cell division cycle 25C. Inhibition of ERK activation through sustained hepatocyte growth factor-induced MDA-MB-231 cell invasion (Tsai et al., 2013; Zhang et al., 2021). Ophiopogonin D significantly inhibits TNBC cell growth and metastasis *in vitro*, mediated in part by inhibition of the integrin subunit beta 1, focal adhesion kinase, Src, AKT, β-catenin signaling pathway (Zhu et al., 2020).

### Immune Effect of Polysaccharides TCM on TNBC

Polysaccharides isolated from TCM have immunomodulatory and antitumor effects and play an important role in immunosuppression (Chen et al., 2006; Liu et al., 2018). Based on the basis of network-based pharmacology it was determined that astragalus polysaccharide may interfere with the invasion and proliferation of MDA-MB-231 cells by inhibiting PI3KCG/AKT/Bcl2 pathway expression and can promote apoptosis. *In vitro* experiments it confirmed that astragalus polysaccharide inhibits TNBC symptoms in a dose-dependent manner (Liu et al., 2019b). Huaier polysaccharide inhibited the stem cell-like characteristics of MDA-MB-231, MDA-MB-453 and Hs578T cells *in vitro* and *in vivo*, partly through the estrogen receptor α-36 signaling pathway (Hu et al., 2019). Fucoidan inhibits MAPK and P13K activation, suppresses activating protein-1 and NF-κB signaling, downregulates pro-angiogenic factor expression in TNBC cells, exhibits anti-ataxia telangiectasia mutated, cell cycle checkpoint kinase 2 proliferative activity against MDA-MB-231 and HCC1806 cells, and effectively reduces migration and invasion (Hsu et al., 2013; Hsu et al., 2020). Cordyceps polysaccharides effectively inhibited MDA-MB-231 cell metastasis and restored drug sensitivity in topotecan-resistant cells by downregulating the transforming growth factor-β signaling pathway and EMT program (Lin et al., 2016).

Shiitake mushroom polysaccharide, Ganoderma lucidum polysaccharide, polysaccharides from the roots of platycodon grandiflorum, Safflower polysaccharide and Ginseng polysaccharide all have strong anti-tumor activity (Chihara et al., 1969; Ando et al., 2002; Gao et al., 2003; Yoon et al., 2003). However, studies in TNBC are still relatively few and the immune mechanism of action is unclear, which deserves further development.

### Immune Effect of Other TCM Ingredients on TNBC

In addition to the aforementioned drugs, tubeimu also exhibited *in vitro* inhibition of TNBC cell migration and invasion without significant toxic side effects (Wang et al., 2018). Cantharidin reverses the metastasis of MDA-MB-231 cells by inhibiting pyruvate kinase isozyme type M2 nuclear translocation and disrupting the glucose transporter 1, pyruvate kinase isozyme type M2 glycolytic cycle, leading to the conversion of aerobic glycolysis to oxidation (Pan et al., 2019). More toxic drugs may limit their use *in vivo* and improve *in vivo* safety by developing a novel drug, α**-**amanitin-conjugated trastuzumab, which kills tumor cells while inducing immunogenic cell death, using the HER2 antibody trastuzumab coupled to α**-**amanitin, and cells containing 17p deletion, which are expressed at low levels of HER2 (Li et al., 2021). Bufalin increased the expression of necrosis mediators threonine kinase1 and threonine kinase3, induced MDA-MB-231 cell death and inhibited the growth of both human breast cancer MCF-7 and MDA-MB-231 cells through the reactive oxygen species-mediated RIP1/RIP3/PARP-1 pathway (Li et al., 2018b).

Arctigenin induces prolonged p21 expression and p38-mediated apoptosis-inducing factor-dependent cell death, which enhanced the toxicity of adriamycin in human breast cancer cells. (Lee et al., 2020). Three triterpenes were isolated from cactus, of which compound d2 was identified as betulinic acid, and treatment of MDA-MB-231 cells with betulinic acid-rich cactus resulted in activation of c-Jun N-terminal kinase as well as downregulation of ERK1 (Foo et al., 2016). Citrus hystrix DC extract and its compounds citronellol and citronellal induce apoptosis in MDA-MB-231 by inhibiting the anti-apoptotic protein Bcl-2, leading to activation of the pro-apoptotic Bcl2-associated X protein and inducing a downstream cystatin-dependent apoptotic pathway by activating cystatin-3 protein (Ho et al., 2020). Rhubarb acid derivatives are new anthraquinones that downregulate Rac1 expression and maybe small molecule inhibitors of Rac1 (Li et al., 2020). Gambogic acid significantly inhibits viability and increases apoptosis of paclitaxel-resistant MDA-MB-231 cells through activation of the sonic hedgehog signaling pathway (Wang et al., 2019). In a word, we have summarized some key ingredients from the above in table 1.

**TABLE 1:** Role of TCM in TNBC.

Compound	Detailed activity/mechanism of action	Application	References
Sinomenine	Reduced human IL-8 mRNA expression in MDA-MB-231 cells; downregulated CXCR1.	*In vivo* and *in vitro*	Zhang et al. (2019)
	ROS-dependent and non-dependent pathways; upregulated the expression of MAPKs.	*In vivo* and *in vitro*	Li et al. (2014)
Berberine	Inhibited IL-1α, IL-1β, IL-6, TNF-α expression; downregulated EGFR protein expression	*In vivo* and *in vitro*	Zhao and Zhang, (2020)
Theophylline	Reduced extracellular levels of HMGB1 protein and downregulated HMGB1 and MMP-9 mRNA expression	*In vitro*	(Wild et al., 2012, Hashemi-Niasari et al. (2018)
Honokiol	Sensitized doxorubicin-resistant breast cancer cells to doxorubicin-induced apoptosis	*In vitro*	Yi et al. (2021)
	Increased solubility of honokiol in nano-micellar formulations	*In vitro* and *in vivo*	Godugu et al. (2017)
	Downregulated the expression and phosphorylation of c-Src, EGFR and AKT; inactivated mTOR and its downstream signal molecules	*In vitro*	Park et al. (2009)
Puerarin	Abrogated the NF-κB; inhibited phosphorylation of p65 and IkBα	*In vitro*	Liu et al. (2017)
	Downregulated ROS production in the activated myofibroblast	*In vivo* and *in vitro*	Xu et al. (2020)
Licorice flavonoids	Reduced MAPK and AKT signaling, suppressed MDA-MB-231 cell migration and invasion	*In vitro*	Huang et al. (2019)
(-)-Epigallocatechin 3-gallate	Reduced the expression of ER-a36 in MDA-MB-231 and MDA-MB-436 cells	—	Pan et al. (2016)
Huaier polysaccharide	Reduced ERα-36 experssion; attenuatted ERα-36-mediated activation of ERβ/α-catenin signaling	*In vivo* and *in vitro*	Hu et al. (2019)
Ginsenoside panaxatriol	Resensitized TNBC paclitaxel resistant cells to pentatonix by inhibiting the IRAK1/NF-κB and ERK pathways	*In vitro*	Wang et al. (2020)
Ginsenoside Rh2	Upregulated p-p53, p-p38, and phospho-ASK1 proteins, downregulated levels of TNF receptor associated factor 2	*In vitro*	Ren et al. (2018)
Ginsenoside Rg3	Regulated Bax/Bcl-2 expression on TNBC by inhibited NF-kB signaling	*In vivo* and *in vitro*	Yuan et al. (2017)
Polyphyllin VI	Inhibited the metastatic potential of 4T1 and MDA-MB-231 cells; attenuated the migration of miR-18a mimic or Rell2 siRNA-enhanced MDA-MB-231 cells	*In vivo* and *in vitro*	Wang et al. (2019)
Timosaponin AIII	Activated ATM/Chk2 and p38 pathways upregulated phospho-histone H2A.X and p-p38 levels	*In vivo* and *in vitro*	Zhang et al. (2021)
	Inhibited the activation of cytosolic activated transcription factor 2; downregulated cyclo-oxygenase-2 and MMP-9 transcription	*In vitro*	Tsai et al. (2013)
Fucoidan	Inhibited TNBC invasiveness and pro-angiogenesis; regulated EMT by modulated TGFR/Smad dependent signaling	—	Hsu et al. (2020)
Cordyceps polysaccharides	Inhibited breast cancer metastasis and restored drug sensitivity in drug-resistant cells by down-regulating the transforming growth factor-β signaling pathway and EMT program	*In vivo* and *in vitro*	Lin et al. (2016)
Bufalin	Inhibited human breast cancer tumorigenesis by inducing cell death through the ROS-mediated RIP1/RIP3/PARP-1 pathways	*In vivo* and *in vitro*	Li et al. (2018)
Arctigenin	Enhanced DOX-induced DNA damage, decreased the phosphorylation of signal STAT3 and the expressions of RAD51 and survivin	*In vitro*	Lee et al. (2020)
Exaction of Citrus hystrix DC (Citronellol and Citronellal)	Induced apoptosis in MDA-MB-231 cells through inhibition of anti-apoptotic Bcl-2 expression, leading to activation of the caspase-3-dependent pathway	*In vitro*	Ho et al. (2020)
Rhubarb acid derivatives	Induced apoptosis, G2/M phase cell cycle arrest and oxidative stress in MDA-MB231 cells; activated pro-apoptotic JNK1; downregulated anti-apoptotic ERK1 and anti-apoptotic bcl-2; increased the bax/bcl-2 ratio and initiated the mitochondrial apoptosis pathway	*In vitro*	Li et al. (2020)

## Conclusion

TCM is widely used as an adjunct method in tumor treatment. Numerous studies have demonstrated that TCM exerts its immune effects through the following pathways, enhanced immune response, reduced immune tolerance, and suppressed tumor immunity. In addition to regulating tumor microenvironment-related factors, such as interleukins, interferons, tumor necrosis factors, chemokines, and growth factors, TCM treatments can also regulate immune cells by restoring the antigen expression function of dendritic cells, enhancement of natural killer cell activity, and inhibition of associated fibroblasts (Takei et al., 2004; Mu et al., 2016; Wang et al., 2020). The above study provides a new therapeutic option for TCM immunotherapy against TNBC.

This paper mainly reviews the immunomodulatory mechanism of different active ingredients of TCM on TNBC. But research on the effects of TCM on immune responses to TNBC is rather scarce. TCM immunomodulatory effects are less studied and there is little information on immune targets. The majority of the studies are focused on immune factor expression. In TCM treatments with demonstrated toxic side effects, exploring how these can be reduced while increasing immune activation against tumors, is worth further investigation. The mechanisms of TCM synergistic radiotherapy drugs on TNBC are still unclear, and the synergistic immune mechanism of multiple drugs still needs to be explored.
